# Inhibition on Proteasome β1 Subunit Might Contribute to the Anti-Cancer Effects of Fangchinoline in Human Prostate Cancer Cells

**DOI:** 10.1371/journal.pone.0141681

**Published:** 2015-10-29

**Authors:** Dong Li, Yu Lu, Peng Sun, Li-Xing Feng, Miao Liu, Li-Hong Hu, Wan-Ying Wu, Bao-Hong Jiang, Min Yang, Xiao-Bo Qu, De-An Guo, Xuan Liu

**Affiliations:** 1 Shanghai Institute of Materia Medica, Chinese Academy of Sciences, Shanghai 201203, P.R. China; 2 Changchun University of Chinese Medicine, Changchun 130117, P.R. China; 3 Nanjing Tianyi Bioscience Co. Ltd, Nanjing 210061, P.R. China; National Health Research Institutes, TAIWAN

## Abstract

Fangchinoline is a bisbenzylisoquinoline alkaloid isolated from Radix *Stephaniae tetrandrae* S. Moore. Fangchinoline and its structure analogue, tetrandrine, exhibited direct binding affinity with recombinant human proteasome β1 subunit and also inhibited its activity *in vitro*. In cultured prostate PC-3 cells and LnCap cells, fangchinoline could dose-dependently inhibit cell proliferation and caspase-like activity of cellular proteasome which was mediated by proteasome β1 subunit. The inhibitive effect of fangchinoline on caspase-like activity of proteasome was also observed in purified human erythrocyte 20S proteasome. In PC-3 cells, fangchinoline induced cell cycle arrest at G0/G1 phase and apoptosis. Treatment of PC-3 tumor-bearing nude mice with fangchinoline inhibited tumor growth, induced apoptosis and also caused decrease in proteasome activities in tumor xenografts. Dose-dependent and time-dependent accumulation of ubiquitinated proteins and important proteasome substrates such as p27, Bax and IκB-α were observed in fangchinoline-treated cells. Over-expression of proteasome β1 subunit by plasmid transfection increased sensitivity of cells to the cytotoxicity of fangchinoline while knockdown of proteasome β1 subunit ameliorated cytotoxicity of fangchinoline in PC-3 cells. Results of the present study suggested that proteasome inhibition was involved in the anti-cancer effects of fangchinoline. Fangchinoline and its structure analogues might be new natural proteasome inhibitors targeting β1 subunit.

## Introduction

**Fig 1:**
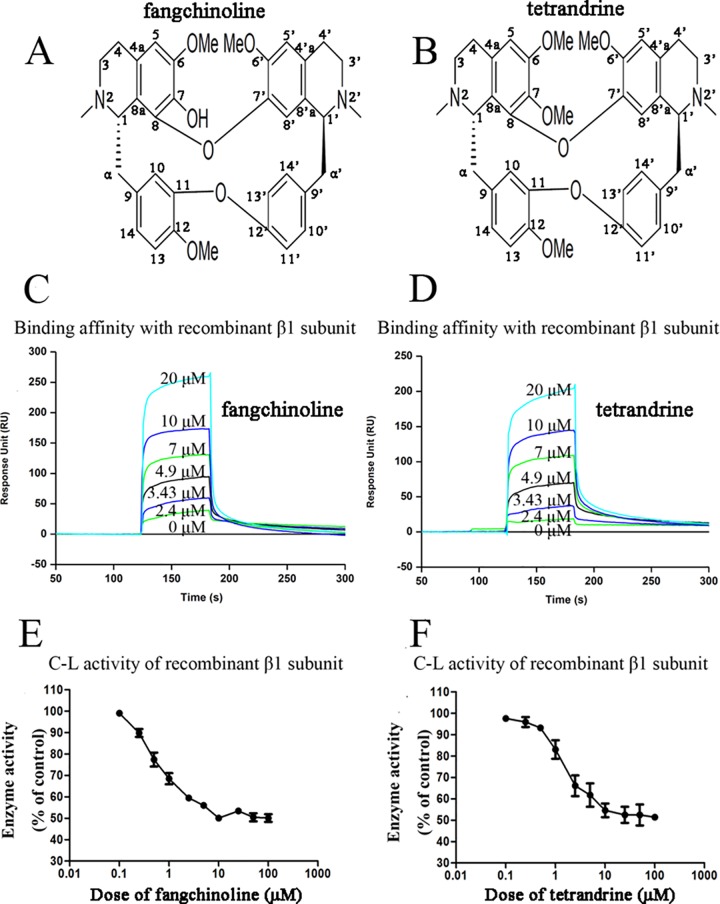
Direct effects of fangchinoline and tetrandrine on recombinant human proteasome β1 subunit. (A) Chemical structure of fangchinoline. (B) Chemical structure of tetrandrine. (C) Real time binding affinity measurements of fangchinoline to the recombinant proteasome β1 subunit protein. (D) Real time binding affinity measurements of tetrandrine to the recombinant proteasome β1 subunit protein. (E) Enzyme activities of recombinant proteasome β1 subunit with or without presence of fangchinoline at different concentrations. (F) Enzyme activities of recombinant proteasome β1 subunit with or without presence of tetrandrine at different concentrations. Data were statistical results of three independent experiments.

Proteasome has emerged as an important and effective target for anti-cancer therapy[1]. In our screening of natural proteasome inhibitors, fangchinoline and tetrandrine, two bisbenzylisoquinoline alkaloids isolated from dried root of *Stephaniae tetrandrine* S. Moore (family, Menispermaceae), known as Fangji in China, were found to have direct binding affinity with recombinant human proteasome β1 subunit and also inhibited its activity *in vitro*. Fangji is a traditional Chinese medicine (TCM) which had been used in clinic as an analgesic, antirheumatic, and antihypertensive drug for a long time in China[2, 3]. Reported activities of Fangji included anti-cancer effects[2, 4], anti-inflammatory effects[5], cardiovascular effects[6, 7], and etc. The main active components of Fangji had been found to be fangchinoline and tetrandrine [2]. The chemical structures of fangchinoline and tetrandrine were shown in Fig 1A and Fig 1B, respectively. Purified tetrandrine had been admitted by China Food and Drug Administration to be used in clinic for treatment of inflammatory diseases such as silicosis[8] and was also used in treatment of cancer[9]. As far as we know, neither any purified compound isolated from Fangji nor crude extract of Fangji had been reported before to have proteasome-inhibiting effects. Proteasome is known to play roles in development of cancer, inflammatory diseases and cardiovascular diseases[10]. Therefore, it might be interesting to study whether proteasome inhibition is involved in the effects of fangchinoline, tetrandrine as well as Fangji.

Both fangchinoline[2, 11–14] and tetrandrine[4, 15–26] had been reported to have anti-cancer effects *in vitro* and *in vivo*. In the present study, we focused on studying the anti-cancer effects of fangchinoline. Previous reports showed that, fangchinoline could induce cell cycle arrest[11, 13, 14, 27] and apoptosis[2, 14, 27] in cultured cancer cells. There was also one report about anti-cancer activities of fangchinoline *in vivo*[13]. In the present study, the anti-cancer effects of fangchinoline were evaluated in cultured PC-3 cells, cultured LnCap cells and PC-3 tumor-bearing nude mice. Both the inhibiting effects of fangchinoline on activities of cellular proteasome in cancer cells and on activities of purified human 20S proteasome were checked. The efficiency of proteasome as a target for cancer therapy was based on the fact that the ubiquitin proteasome system was responsible for the degradation of many important intracellular proteins involved in critical cellular processes such as cell cycle progression, proliferation and apoptosis[28–30]. Therefore, the effects of fangchinoline treatment on the degradation of important proteasome substrates such as p27 which played role in cell cycle progression, Bax which was involved in apoptosis, and IκB which related to NF-κB pathway[31] were further checked in the present study. Furthermore, we also observed the influence of over-expression of proteasome β1 subunit or knockdown of proteasome β1 subunit on sensitivity of cells to the cytotoxicity of fangchinoline.

Collectively, our results suggested the involvement of proteasome inhibition in the anti-cancer effects of fangchinoline *in vitro* and *in vivo*. Results of the present study contribute to understanding the anti-tumor mechanism of fangchinoline as well as Fangji.

## Materials and Methods

### Chemicals

Fangchinoline with a purity more than 98% and tetrandrine with a purity more than 98% were both purchased from Shanghai Source Leaf Technology Co., Ltd. (Shanghai, China). Fangchinoline or tetrandrine was dissolved in DMSO to the concentration of 0.1 M as stock solution and stored at -20°C. Fluorogenic peptide substrates Z-LLE-AMC for checking proteasome caspase-like (C-L) activity and Z-ARR-AMC for checking proteasome trypsin-like (T-L) activity were from Calbiochem, Merck. Fluorogenic peptide substrate Suc-LLVY-AMC for checking proteasome chymotrypsin-like (CT-L) activity was from Sigma-Aldrich. Other chemical reagents, except where specially noted, were purchased from Sigma-Aldrich.

### Cell culture

Human PC-3 prostate cancer cells and human LnCap prostate cancer cells were purchased from Cell Resource Center of Shanghai Institutes for Biological Sciences, Chinese Academy of Sciences. PC-3 cells and LnCap cells were cultured in RPMI 1640 medium supplemented with 10% fetal bovine serum, 100 units/mL of penicillin, and 100 μg/mL of streptomycin and maintained at 37°C and 5% CO_2_. Fetal bovine serum, RPMI 1640, penicillin and streptomycin were all from Hyclone.

### Surface plasmon resonance biosensor analysis

The recombinant human proteasome β1 subunit, product of PSMB6 gene, was expressed as His-Tag proteins using Escherichia coli, and then purified by affinity chromatography as reported in our previous study[32]. The binding affinities of fangchinoline or tetrandrine to recombinant proteasome catalytic β1 subunit, were assayed *in vitro* using an Biacore 3000 instrument (Biacore AB, Rapsgatan 7, S-754 50 Uppsala, Sweden) as reported before[33]. Briefly, recombinant proteasome β1 subunit protein was immobilized on a CM5 sensor chip as ligand in 11624.5 RU with N-ethyl-N’-(3-dimethylaminopropyl) carbodiimide and N-hydroxysuccinimide according to the standard primary amine-coupling procedures, and HBS-EP (10 mM HEPES, 150 mM NaCl, 3 mM EDTA, 0.005% (v/v) surfactant P20, pH 7.4) was used as the running buffer. Equilibration of the baseline was performed by a continuous flow of HBS-EP through the chip surface for 1 to 2 h. Biacore data were collected at 25°C with HBS-EP as the running buffer at a constant flow of 20 μL/min. Fangchinoline or tetradrine was serially diluted into the running buffer to 0, 2.4, 3.43, 4.90, 7.0, 10 and 20 μM. The samples were injected into the channels at a flow rate of 20 μL/min, followed by washing with the running buffer. The binding responses were recorded continuously in response units (RU) at a frequency of 1Hz as sensorgrams and presented as a function of time. The association (*k*_a_) and dissociation (*k*_d_) rate constants, the equilibrium dissociation constant ($KD=\frac{k_{d}}{k_{a}}$) were determined by analysis of the sensorgram-curves obtained at different concentrations of the compound by use of BIA evaluation software version 3.1 (Biacore) and the 1:1 Langmuir binding fitting model.

### Assay of enzyme activity of recombinant proteasome catalytic β1 subunit

Briefly, catalytic activity of the recombinant human proteasome β1 subunit was assayed by adding recombinant β1 subunit protein (30 μg) to 100 μL of proteasome activity assay buffer (10 mM Tris-HCL, pH 7.8, 5 mM adenosine triphosphate, 0.5 mM dithiothreitol and 5 mM MgCL_2_•6H_2_O) containing 50 μM Z-LLE-AMC in the presence of fangchinoline or tetrandrine at different concentrations or not. The hydrolase activity of the proteasome catalytic β1 subunit could release the fluorogenic AMC component from substrate Z-LLE-AMC and the AMC release was measured after 2 h incubation using a Microplate Reader Bio-Rad 550 with excitation and emission wavelengths of 360 and 465 nm, respectively.

### MTT assay of effects of fangchinoline on proliferation of PC-3 cells and LnCap cells

The inhibitive effects of fangchinoline on proliferation of PC-3 cells or LnCap cells was observed by checking cell viability of cells using MTT assay as described before[33]. Briefly, cells were seeded in 96-well plates at a density of 5×10^4^ cells/mL for PC-3 cell and 4×10^4^ cells/mL for LnCap cells. After overnight culture, the media were changed into fresh media containing various concentrations of fangchinoline or 0.1% DMSO (solvent control) for 24, 48 h or 72 h. Cell viability was evaluated by measuring the mitochondrial-dependent conversion of the yellow tetrazolium salt MTT to purple formazan crystals by metabolic active cells. The optical density (proportional to the number of live cells) was assessed with a Microplate Reader Bio-Rad 550 at 570 nm. IC_50_ value (half-maximal inhibitory concentration) was calculated by the Logit method.

### Assay of cellular proteasome activities of PC-3 cells and LnCap cells

Cells were treated with either solvent control (0.1% DMSO), fangchinoline at different concentrations, or 1 μM carfilzomib (positive control) for 24 h at 37˚C. The enzymatic activities of cellular proteasome in cell lysate were measured as reported in our previous paper[32]. Briefly, cells were harvested, washed with PBS and then lysed in proteasome activity assay buffer for 30 min at 4°C. The homogenate was then centrifuged at 12000 × g for 30 min at 4°C. The supernatant was collected as whole cell extract and the protein content in the supernatant was measured with the Bradford reagent. Catalytic activities of cellular proteasome were assayed by adding whole cell extract (containing 30 μg protein) to 100 μL of proteasome activity assay buffer containing 50 μM fluorogenic peptide substrates such as Z-LLE-AMC for detecting C-L activity, Z-ARR-AMC for detecting T-L activity or Suc-LLVY-AMC for detecting CT-L activity, respectively. The release of fluorogenic AMC from the peptide substrates was then measured as described above.

### Assay of C-L activity of purified human 20S proteasome

The 20S proteasome assay kit was bought from Enzo Life Sciences, Inc. (Farmingdale, NY 11735, USA). The K_m_, V_max_ values of the purified human erythrocyte 20S proteasome for fluorigenic peptide substrate Z-LLE-AMC are measured according to the instructions provided by the manufacturer. Inactivation of purified human erythrocyte 20S proteasome by fangchinoline was determined by monitoring the hydrolysis of substrate in the presence of different concentrations of fangcinoline. Assays were performed at 37°C in a reaction buffer (50 μL) containing 50 mM Tris/HCL, pH 7.5, 25 mM KCL, 10 mM NaCL, 1 mM MgCL_2_, 0.1 μg 20S proteasome and Z-LLE-AMC. Reactions were allowed to proceed for 120 min, and fluorescence data were collected every 1 min, and then plotted as a function of time. The apparent dissociation constant, K_i_^app^, was determined by non-linear least-fit of the fractional velocity, v_i_/v_0_, as a function of [I], where v_i_ is the steady state residual activity of the enzyme in the presence of inhibitor (I) and v_0_ is the initial velocity in the absence of inhibitor ($\frac{V_{i}}{V_{0}}=\frac{1}{1+\frac{\left[I\right]}{K_{i}^{\mathrm{app}}}}$). The dissociation constant, Ki, was calculated from the apparent dissociation constant, K_i_^app^, using the following expression, where [S] is the substrate concentration and K_m_ is the substrate binding constant ($K_{i}=\frac{K_{i}^{\mathrm{app}}}{1+\frac{\left[S\right]}{K_{m}}}$).

### Induction of cell cycle arrest and apoptosis by fangchinoline in PC-3 cells

The induction of cell cycle arrest and apoptosis by fangchinoline in PC-3 cells was observed by using flow cytometric analysis as described in our previous reports [32–34]. Briefly, for flow cytometry analysis of cell cycle, cells were collected after treatment, washed with PBS and then fixed with 70% ethanol at 4°C for overnight. After PBS washes, cells were re-suspended in staining buffer (10 mg/mL RNase A and 5 mg/mL propidium iodide in cold PBS) for 30 min in dark at room temperature and then analyzed using FACSCalibur Flow Cytometer. For flow cytometric analysis of apoptosis, Alexa Fluor 488 Annexin V & PI /Dead cell Apoptosis Kit (Invitrogen) was used. Cells were collected after treatment, washed with PBS and then resuspended in binding buffer and then incubated with Annexin V-FITC and propidium iodide for 15 min in dark at room temperature. Then, flow cytometric analysis was conducted and data analysis was performed with CellQuest software.

### PC-3 tumor xenograft experiments

Male nude immunodeficient mice Balb/c-nu-nu, aged 4 weeks, were purchased from Shanghai Experimental Animal Center. All procedures involving animals were approved by the Institutional Animal Care and Use Committee of Shanghai Institute of Materia Medica, Chinese Academy of Sciences. On day 0, PC-3 cells (8×10^6^ cells) suspended in 0.2 mL of serum-free RPMI 1640 were inoculated s.c. in the right flank of each mouse (six mice per group). 14 days after inoculation, mice were randomly divided into 3 groups and started injection with either vehicle control or fangchinoline at 25 mg/kg (ip., QD) or 50 mg/kg (ip., QD). Tumor sizes were measured every 4 days using calipers and their volumes were calculated using a standard formula: width^2^×length/2. The relative tumor volume (RTV) was defined as the ratio of the tumor volume at a indicated time and the tumor volume at the start of drug treatment. Body weight was measured daily. On day 30 after inoculation, mice were sacrificed by CO_2_ inhalation and tumor xenografts were dissected. To visualize apoptosis in tumor xenografts, TUNEL labeling was conducted to detect apoptotic nuclei using In Situ Death Detection Kit (Roche Molecular Biochemicals). Briefly, tumor xenografts were fixed with 10% formalin solution at room temperature for 24 h and then embedded by paraffin. Specimens were heated for 1h by Water Bath-Slide Drier (LEICA H1 1220, German), incubated with dimethylbenzene for two times (8 min each) and then incubated with 100%, 95%, 90% and 85% ethanol for two times (3 min each). Specimens were then incubated with 0.1M Citrate buffer (pH 6.0) for microwave irradiation. After washing with PBS for two times (3 min each), slices were incubated with reaction buffer (from the In Situ Death Detection Kit) in a humid atmosphere at 37°C for 1 h. Slices were then washed with PBS three times (3 min each) to remove unincorporated fluorescein–deoxyuridine triphosphate. Nuclei were counterstained with 4,6-diamidino-2-phenylindole (DAPI) 1 μg/mL for 3 min and then washed with PBS for three times (3 min each). Images were captured using a 20X objective (Olympus BX51, Japan) on an epifluorescence microscope. Furthermore, the proteasome activities of tumor xenografts were assayed. Briefly, tumor xenografts were homogenerized in proteasome activity assay buffer using the FastPrep System (MP FastPrep-24, U.S.A), then centrifugation at 4°C for 30 min. The supernatants were then used for assay of proteasome activities using method as described above for analysis of cellular proteasome activities.

### Western blotting analysis

Western blotting assay was conducted as described before[33, 34]. Briefly, an aliquot of protein (100 μg) sample was loaded onto a 12% SDS gel, separated electrophoretically, and transferred to a Nitrocellulose membrane (Bio-Rad). After the membrane was incubated with 10 mM TBS with 20% Tween 20 and 5% dehydrated skim milk to block nonspecific protein binding, the membrane was incubated with primary antibodies overnight at 4°C. The primary antibodies (all from Cell Signaling Technology) used were rabbit polyclonal antibody against human IκB-α (#4812, 1:400), Bax (#2772, 1:400), proteasome β1 subunit (PSMB6) (#13267, 1:500), β-actin (#4967, 1:400) and mouse monoclonal antibody against human p27 Kip1 (#3688, 1:400) or ubiquitin (#3936, 1:1000). After TBS washes, blots were then incubated with secondary antibody for 2 h at room temperature at a 1:1000 dilution and then visualized using chemiluminescence (Pierce Biotechnology, Rockford, IL, USA). The secondary antibodies used were HRP-linked goat anti-rabbit IgG (#7074) and HRP-linked horse anti-mouse IgG (#7076), both from Cell Signaling Technology.

### Over-expression of proteasome β1 subunit in PC-3 cells by plasmid transfection

Cells with over-expression of proteasome β1 subunit were obtained by transfection with pcDNA3.1 plasmid encoding full-length human PSMB6 cDNA as described before[34]. For plasmid transfection, 5 × 10^5^ cells were seeded into each well of a six-well plate until the cells reached approximately 80% confluence after cultured for 24 h. 2.5 μg pcDNA3.1 plasmid DNA encoding PSMB6 or pcDNA3.1 plasmid DNA without PSMB6 (as negative control) was diluted in 250 μL serum-free medium and then incubated for 20 min at room temperature with a mixture of 10 μL Lipofectamine 2000 (Invitrogen) and 400 μL serum-free medium. The resultant complex mixture was then added to each well of the cell culture plate. After 6 h incubation, the complex medium was replaced with fresh minimum essential medium supplemented with 10% (v/v) FBS and let to grow overnight. Then, G418 (Merck) was added to the medium to reach a final concentration equal to the minimum fatal dose (400 μg/mL). The cells were allowed to grow and passage in the presence of G418 for over 2 weeks. Expression of proteasome β1 subunit in the transfected cells was checked using Western blotting assay and compared with that of wild-type cells and negative-control cells. The cytotoxicity of fangchinoline on cells with over-expression of proteasome β1 subunit was then checked using MTT assay as described above and compared with that of wild-type cells and negative-control cells.

### Knockdown of proteasome β1 subunit in PC-3 cells by siRNA transfection

Validated siRNAs for PSMB6 (Sigma-Aldrich) were transfected into PC-3 cells by using Lipofectamine RNAiMAX (Invitrogen) according to the manufacturer’s protocol. Scrambled negative control siRNAs (Sigma-Aldrich) were used as negative control. Expression levels of PSMB6 (proteasome β1 subunit) in wide type cells, PSMB6 knockdown cells (cells treated with siRNAs for PSMB6), negative control cells (cells transfected with scrambled negative control siRNAs) were checked using Western blotting assay. To check the influence of proteasome β1 subunit knockdown on cytotoxicity of fangchinoline, cells were transfected with siRNAs for PSMB6 or scrambled negative control siRNAs for 24 h. Then, cells were seeded into 96-well plates at a density of 5 × 103 cells/well and the effects of fangcinoline on cell proliferation were checked using MTT assay as described above. Furthermore, the effects of fangchinoline on markers of apoptosis and autophagy in cells with normal or knockdown of β1 subunit were checked using Western blotting assay as described above. Briefly, after transfected with siRNAs for PSMB6 or scrambled negative control siRNAs for 24 h, cells were treated with fangchinoline at 40 μM for 24 h. Then, the cells were harvested for Western blotting assay. The primary antibodies used were rabbit anti-caspase 3 antibody (1:1000, Cell Signaling Technology, Cat#9662), rabbit anti-PARP antibody (1:1000, Cell Signaling Technology, Cat#9542) and rabbit anti-LC3B antibody (1:1000, Cell Signaling Technology, Cat#2775). The secondary antibody used was HRP-linked goat anti-rabbit IgG (1:1000, Cell Signaling Technology, Cat#7074).

### Statistical analysis

Student’s *t*-test was applied to evaluate the differences between treated and control groups. Data were expressed as mean ± SEM and results from at least 3 independent experiments were used for statistical analysis. Asterisks indicate a significant difference (P < 0.05) compared with un-treated control.

## Results

### Binding affinities and direct inhibitive effects of fangchinoline/tetrandrine on recombinant proteasome β1 subunit

Both fangchinoline (Fig 1C) and tetrandrine (Fig 1D) exhibited direct binding affinity with recombinant human proteasome β1 subunit. As shown in Fig 1C, RU increased with increasing fangchinoline concentration, which indicated that fangchinoline was able to bind to proteasome β1 subunit in a dose-dependent manner. Similar results were observed for tetrandrine (Fig 1D). The association (*k*_a_), dissociation (*k*_d)_ and equilibrium dissociation (*K*_D_) constants of fangchinoline or tetrandrine binding to the proteasome β1 subunit were shown in Table 1. The results suggested that fangchinoline and tetrandrine exhibited similar binding affinity to proteasome β1 subunit. Furthermore, both fangchinoline and tetrandrine could directly inhibit the enzyme activity of recombinant proteasome β1 subunit *in vitro*. As shown in Fig 1E, fangchinoline dose-dependently inhibited the activity of recombinant proteasome β1 subunit. Similar results were observed for tetrandrine (Fig 1F).

**Table 1:** Biacore assay results of binding affinity between recombinant human proteasome β1 subunit and fangchinoline or tetrandrine.

Compound	Association (*k*_a_) (M^-1^S^-1^)	Dissociation (*k*_d_) (S^-1^)	Equilibrium dissociation (*K*_D_) (μM)
fangchinoline	7.55×10^3^	0.0353	4.68
tetrandrine	2.89×10^3^	0.0162	5.61

### Inhibitive effects of fangchinoline on proliferation and cellular proteasome activities of cancer cells

**Fig 2:**
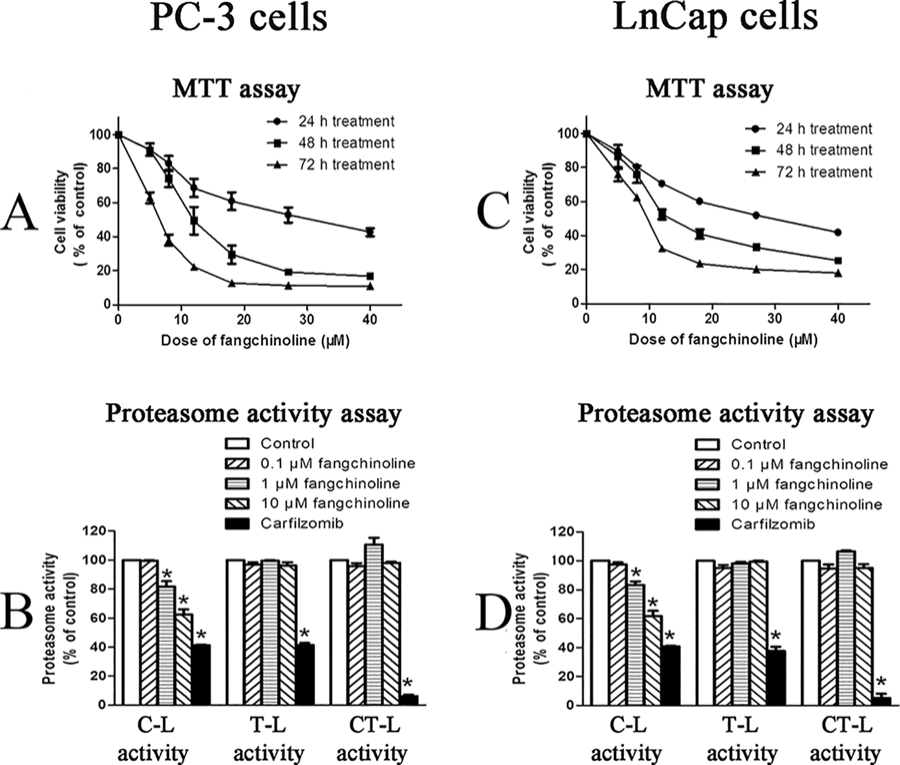
Inhibitive effects of fangchinoline on cell proliferation and cellular proteasome activities of PC-3 cells and LnCap cells. (A) Cell viability (MTT assay result) of PC-3 cells treated with various concentrations of fangchinoline for 24, 48 or 72 h. Data were statistical results of three independent experiments. (B) Cellular proteasome activities of PC-3 cells treated with 0.1% DMSO or fangchinoline at different concentrations for 24 h. (C) Cell viability (MTT assay result) of LnCap cells treated with various concentrations of fangchinoline for 24, 48 or 72 h. (D) Cellular proteasome activities of control PC-3 cells treated with 0.1% DMSO (solvent control) or fangchinoline at different concentrations for 24 h. Data were statistical results of three independent experiments. *p<0.05 vs. solvent control. Carfilzomib was used as positive control.

As shown in Fig 2A, fangchinoline could dose-dependently and time-dependently decrease the viability of PC-3 cells. The IC_50_ values of fangchinoline in inhibiting cell proliferation of PC-3 cells were 27.53±3.346 μM for 24 h, 13.13±1.579 μM for 48 h, and 7.247±0.3122 μM for 72 h treatment, respectively. Furthermore, as shown in Fig 2B, fangchinoline could dose-dependently inhibit the C-L activity of cellular proteasome, which was mediated by proteasome β1 subunit. The inhibition on C-L activity was significant at doses of 1 and 10 μM fangchinoline. The activities of proteasome trypsin-like (T-L) and chymotrypsin-like (CT-L) activities, mediated by proteasome β2 and β5 subunit, were not significantly affected by fangchinoline treatment.

Similar results were observed in another cancer cell line, LnCap cells. Fangchinoline dose-dependently and time-dependently decrease the viability of LnCap cells (Fig 2C). The IC_50_ values of fangchinoline in inhibiting cell proliferation of LnCap cells were 36.18±6.33 μM for 24 h, 11.59±0.22 μM for 48 h, and 12.73±0.59 μM for 72 h treatment, respectively. And, fangchinoline also could significantly inhibit the C-L activity of cellular proteasome but did not affect the activities of proteasome T-L and CT-L activities in LnCap cells (Fig 2D).

### Inhibitive effects of fangchinoline on C-L activity of purified human 20S proteasome

**Fig 3:**
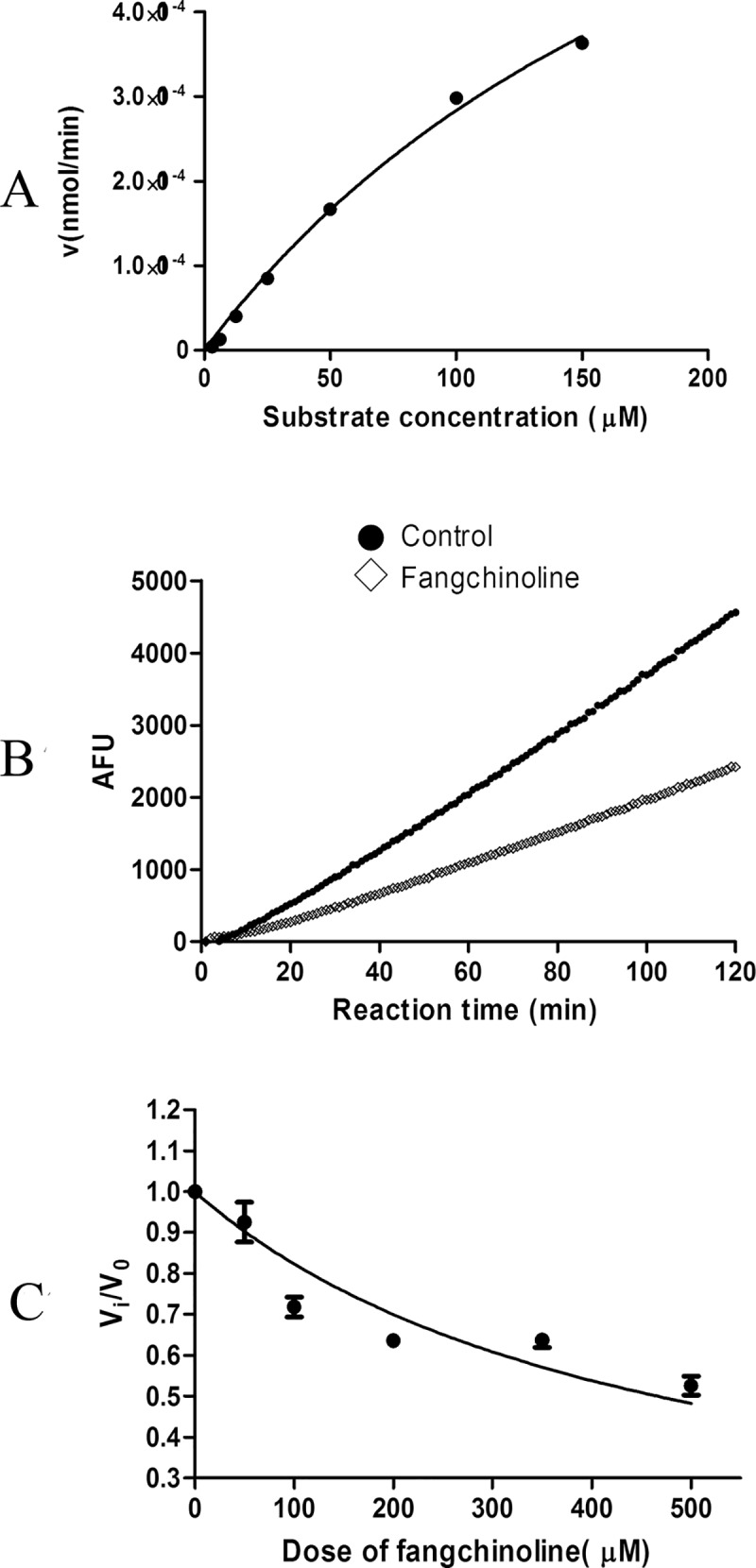
Inhibitive effects of fangchinoline on activity of purified human 20S proteasome. (A) Hydrolysis of the β1-specific fluorigenic peptide substrate Z-LLE-AMC at different concentrations by purified human 20S proteasome. (B) Time-dependent hydrolysis of 75 μM substrate Z-LLE-AMC by 20S proteasome with or without presence of 500 μM fangchinoline. (C) Inhibitive effects of fangchinoline at different concentrations on hydrolysis activity of 20S proteasome. Data were statistical results of three independent experiments.

The hydrolysis curve of β1-specific fluorigenic peptide substrate Z-LLE-AMC at different concentrations by purified human 20S proteasome was shown in Fig 3A. The K_m_ and V_max_ of the purified proteasome was calculated to be 244.2 μM and 9.749X10^-4^ nmol/min, respectively. The representative time-dependent hydrolysis curves of Z-LLE-AMC (75 μM) by proteasome with or without presence of 500 μM fangchinoline were shown in Fig 3B. The results indicated that the hydrolysis of substrate by proteasome was partly inhibited in the presence of fangchinoline. The inhibitive effects of fangchinoline at different doses were shown in Fig 3C. The K_i_ of fangchinoline were calculated to be 356.58±30.63 μM.

### Fangchinoline induced cell cycle arrest and apoptosis in PC-3 cells

**Fig 4:**
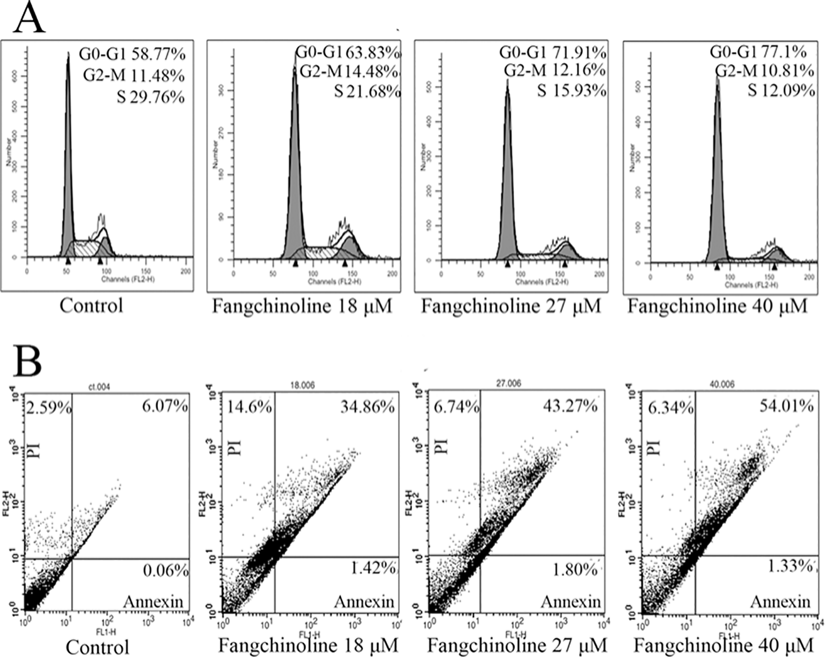
Fangchinoline induced cell cycle arrest at G0/G1 phase and apoptosis in PC-3 cells. (A) Representative flow cytometry analysis results of DNA histograms of PC-3 cells treated with various concentrations of fangchinoline for 24 h. Cell cycle arrest at G0/G1 phase was observed. (B) Representative flow cytometric analysis results of apoptosis in PC-3 cells treated with various concentrations of fangchinoline for 24 h. Showed were representative results of three independent experiments.

Representative results of cell cycle analysis were shown in Fig 4A and statistical analysis results were shown in Table 1. Fangchinoline-treated PC-3 cells appeared to accumulate at the G0/G1 phase with a concomitant decrease in the percentage of cells in the S phase. And, fangchinoline also dose-dependently induced apoptosis in PC-3 cells. Representative results of apoptosis analysis were shown in Fig 4B and statistical analysis results were shown in Table 2.

**Table 2:** Fangchinoline induced cell cycle arrest at G0/G1 phase and apoptosis in PC-3 cells.

Dose of fangchinoline (μM)	Percentage (%) of cells in G0/G1 phase	Percentage (%) of cells in G2/M phase	Percentage (%) of cells in S phase	Percentage (%) of apoptotic cells (Annexin^+^/PI^+^)
0	56.52±1.15	10.29±0.98*	33.20±2.00	4.21±0.93*
5	60.17±1.49	11.21±2.45*	28.62±2.31	6.74±1.14*
8	64.25±1.57*	11.99±2.23*	23.76±2.77	10.79±0.51*
12	63.03±0.73*	11.59±1.99*	25.37±1.75*	13.53±0.19*
18	67.63±3.34*	8.88±2.91	23.48±2.14*	28.10±5.93*
27	75.16±1.95*	8.65±1.81	16.19±0.63*	37.52±4.04*
40	80.14±1.55*	7.99±1.43	11.88±0.56*	49.77±2.23*

*P<0.05 compared with control (dose at 0 μM).

### Fangchinoline treatment inhibited the growth of PC-3 prostate cancer xenografts and induced apoptosis

**Fig 5:**
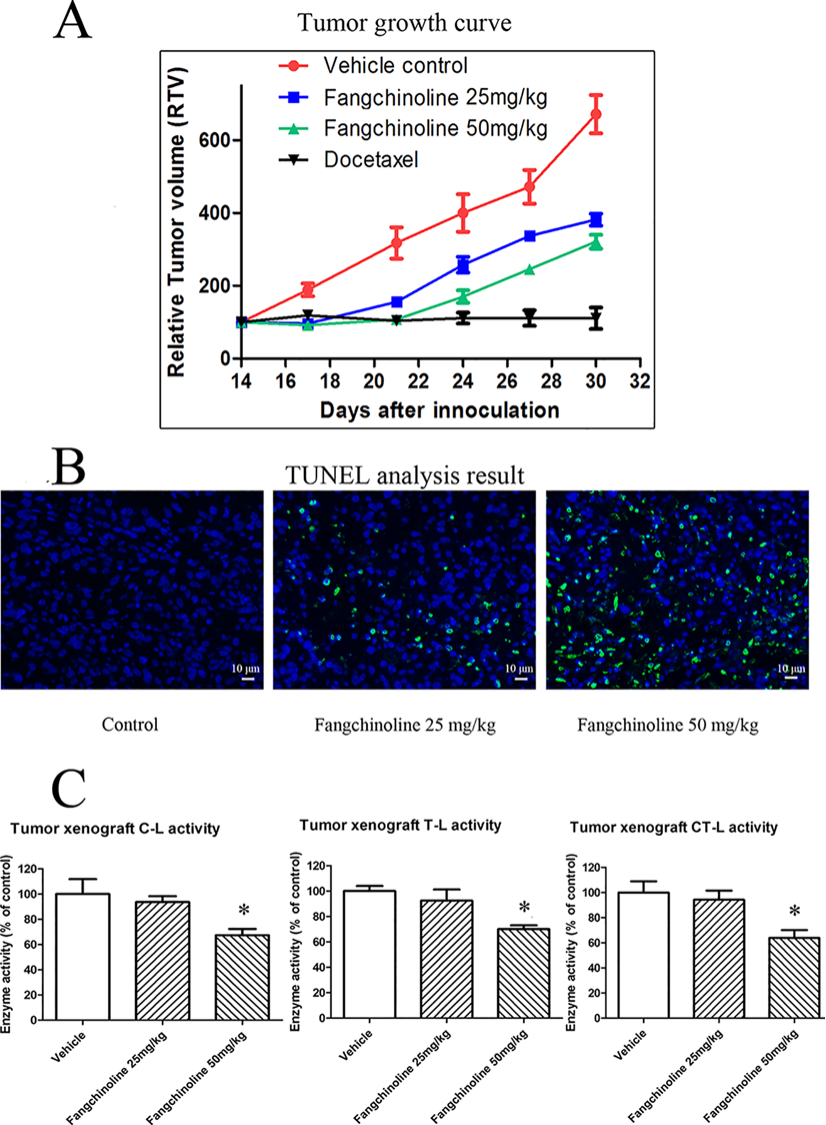
*In vivo* anti-cancer effects of fangchinoline in nude mice inoculating with PC-3 cells. (A) Tumor growth curve in nude mice treated with vehicle control, 25 mg/kg fangchinoline or 50 mg/kg fangchinoline. (B) Apoptosis (showed by TUNEL labeling) induced by fangchinoline in tumor xenografts of animals treated with fangchinoline. Scale bar = 10 μm. (C) Proteasome activities of tumor xenografts of animals treated with vehicle control or fangchinoline. *P<0.05 vs. control group.

As shown in Fig 5A, fangchinoline treatment at 25 and 50 mg/kg could dose-dependently inhibit the growth of PC-3 xenografts. Data showed in Fig 5A was relative tumor volume (RTV) which was defined as the ratio of the tumor volume at a indicated time and the tumor volume at the start of drug treatment. The data of tumor volume (mm^3^) of each group was showed in S1 Table. And, results of TUNEL labeling (Fig 5B) showed that apoptosis was induced in PC-3 xenografts of animals treated with fangchinoline.

### Proteasome inhibition in xenografts of animals treated with fangchinoline

As shown in Fig 5C, results of proteasome activity assay of the tumor xenografts indicated that proteasome activities of xenografts of fangchinoline-treated groups were decreased compared with that of vehicle control. The decrease in proteasome activity was significant in 50 mg/kg fangchinoline-treated group.

### Fangchinoline induced accumulation of ubiquitinated proteins as well as Ub-IκBα, Ub-p27 and Ub-Bax

**Fig 6:**
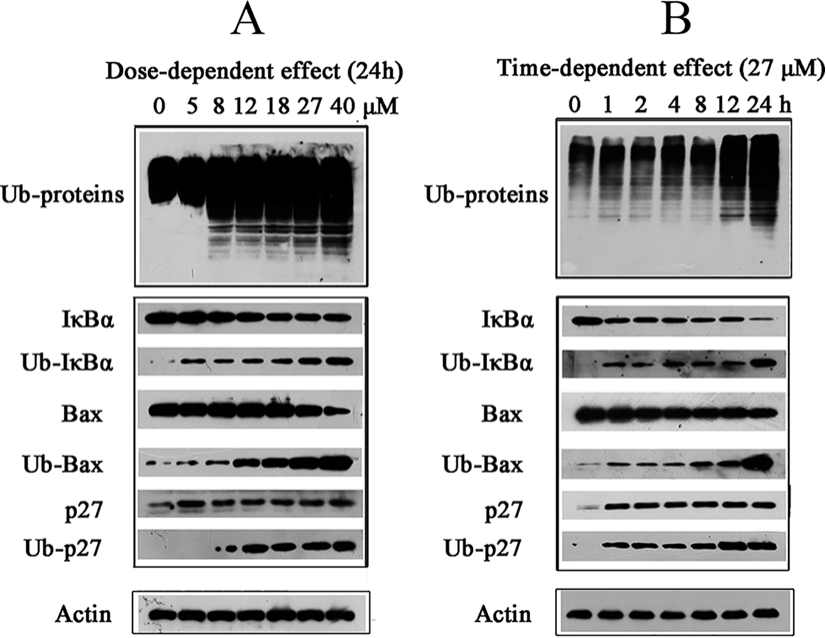
Effects of fangchinoline on accumulation of ubiquitinated proteins. (A) Dose-dependent effects of 24 h fangchinoline treatment on accumulation of ubiquitinated proteins and levels of IκB-α, Bax and p27 in PC-3 cells. (B) Time-dependent effects of 27 μM fangchinoline treatment on accumulation of ubiquitinated proteins and levels of IκB-α, Bax and p27 in PC-3 cells. Showed were representative results of three independent experiments.

Proteasome inhibition would cause accumulation of ubiquitinated proteins. As shown in Fig 6A and Fig 6B, fangchinoline induced dose-dependent and time-dependent accumulation of ubiquitinated proteins in cells. The accumulation of ubiquitinated proteins could be observed at dose as low as 8 μM for 24 h treatment (Fig 6A) or after only 1 h treatment of 27 μM fangchinoline (Fig 6B). The levels of important proteasome target proteins, such as IκBα, Bax, and p27, were also checked in fangchinoline-treated PC-3 cells. The results indicated that fangchinoline dose-dependently and time-dependently induced increase in ubiquitinated IκBα (Ub-IκBα), Ub-p27 and Ub-Bax in PC-3 cells treated with fangchinoline.

### Over-expression of proteasome β1 subunit increased the sensitivity of cells to cytotoxictiy of fangchinoline

**Fig 7:**
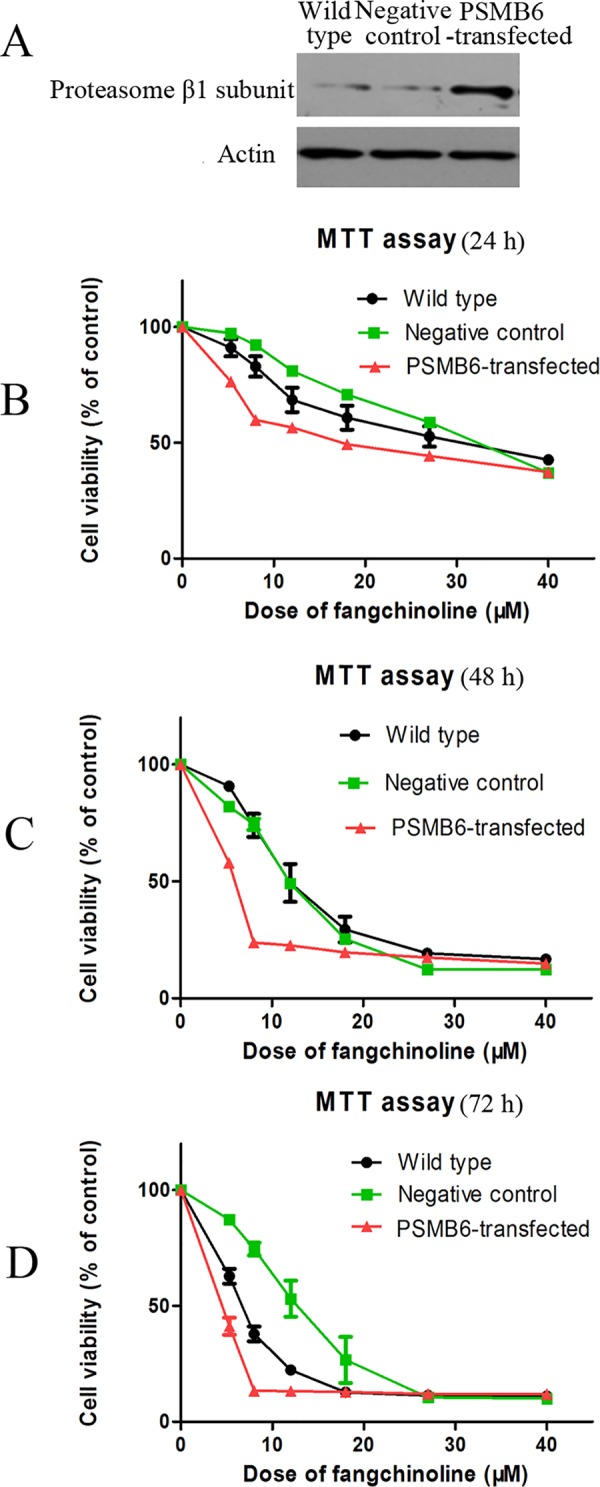
Over-expression of proteasome β1 subunit increased sensitivity of cells to cytotoxicity of fangchinoline. (A) Results of Western blotting assay of proteasome β1 subunit protein expression in wild-type cells, negative-control cells and cells transfected with plasmid encoding PSMB6. (B) Cell viability of wild-type cells, negative-control cells and cells transfected with plasmid encoding PSMB6 after 24 h treatment of fangchinoline at different concentrations. (C) Cell viability of wild type cells, negative control cells and cells transfected with plasmid encoding PSMB6 after 48 h fangchinoline treatment. (d) Cell viability of wild type cells, negative control cells and cells transfected with plasmid encoding PSMB6 after 72 h fangchinoline treatment. Data were statistical results of three independent experiments.

As shown in Fig 7A, transfection of plasmid encoding proteasome subunit beta type 6 (PSMB6) cDNA caused over-expression of proteasome β1 subunit in PC-3 cells. Results of MTT assay of the inhibitive effects of fangchinoline on proliferation of wild type, negative control and PSMB6-transfected cells showed that cells with over-expression of proteasome β1 subunit were more sensitive to cytotoxictiy of fangchinoline treatment for 24 h (Fig 7B), 48 h (Fig 7C) or 72 h (Fig 7D).

### Knockdown of proteasome β1 subunit ameliorated the cytotoxictiy of fangchinoline

**Fig 8:**
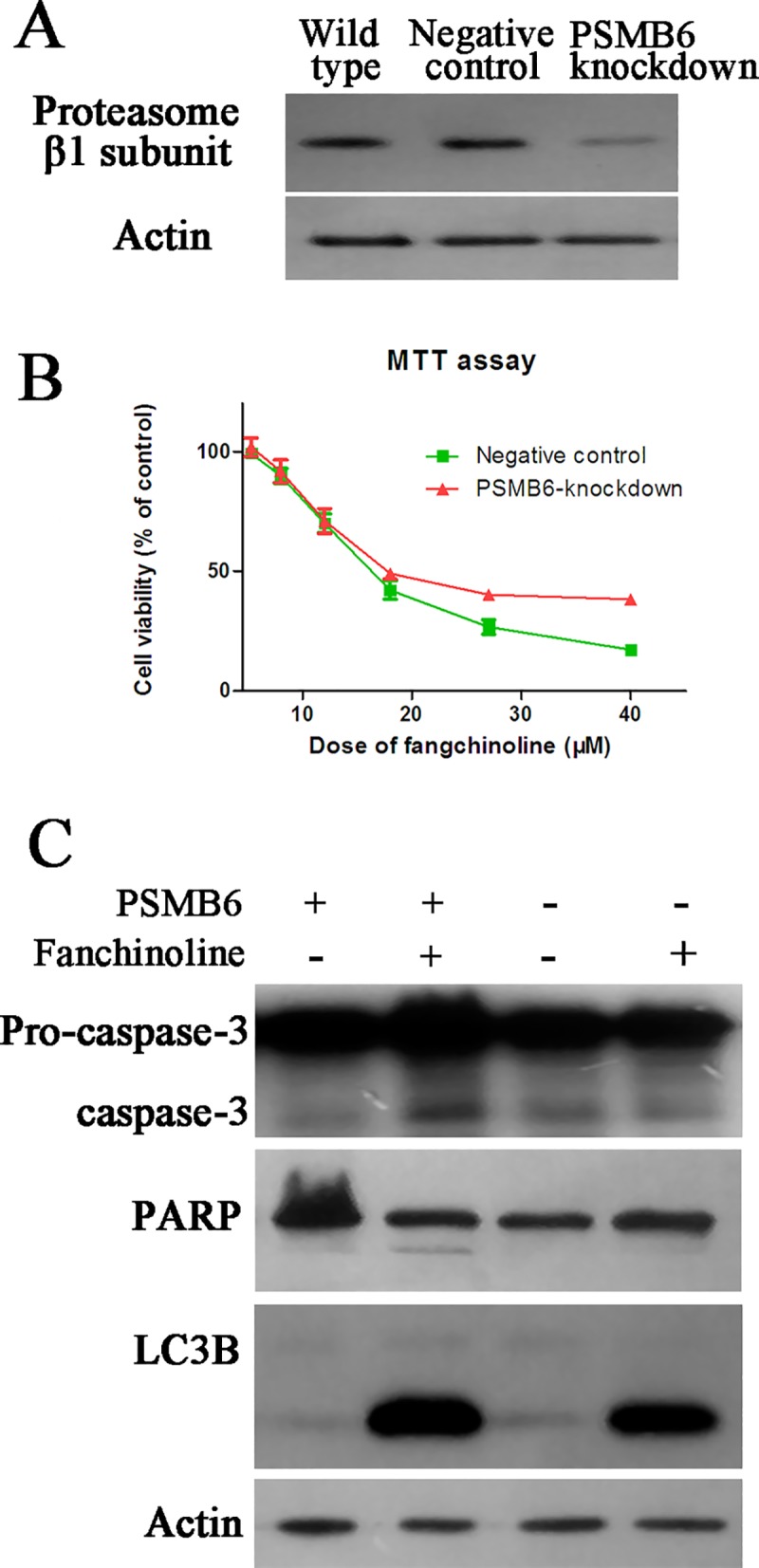
Knockdown of proteasome β1 subunit ameliorated cytotoxicity of fangchinoline in PC-3 cells. (A) Results of Western blotting assay of proteasome β1 subunit protein expression in wild-type cells, negative-control cells and PSMB6 knockdown cells (cells transfected with siRNAs for PSMB6). (B) Cell viability of negative-control cells and cells transfected with siRNAs for PSMB6 after 72 h treatment of fangchinoline at different concentrations. Data were statistical results of three independent experiments. (C) Effects of fangchinoline treatment on markers of apoptosis and autophagy in negative control cells or cells transfected with siRNAs for PSMB6. Showed were representative results of three independent experiments.

As shown in Fig 8A, knockdown PSMB6 expression by siRNA transfection decreased expression of proteasome β1 subunit in PC-3 cells. MTT assay of the effects of fangchinoline on proliferation of negative control and PSMB6-knockdown cells showed that knockdown of proteasome β1 subunit ameliorated proliferation-inhibiting effects of fangchinoline (Fig 8B). Furthermore, Western blotting assay of makers of apoptosis (caspase-3 and PARP) and autophagy (LC-3B) showed that knockdown of proteasome β1 subunit also ameliorated fangchinoline-induced apoptosis and autophagy (Fig 8C).

## Discussion

In the present study, fangchinoline and tetrandrine, bisbenzylisoquinoline alkaloids isolated from Fangji, were demonstrated to have inhibitive effects on proteasome β1 subunit. They could directly bind with recombinant proteasome β1 subunit, inhibit activity of recombinant proteasome β1 subunit *in vitro*. Proteasome β1, β2 and β5 subunits exert caspase-like (C-L), trypsin-like (T-L) and chymotrypsin-like (CT-L) activity, respectively. Fangchinoline could significantly inhibit the C-L activity of cellular proteasome, mediated by proteasome β1 subunit, in both PC-3 cells and LnCap cells. Interestingly, the IC_50_ values of fangchinoline in inhibiting cell proliferation was similar to that of its inhibiting effects on cellular proteasome. For example, the IC50 value of fangchinoline in inhibiting cell proliferation was 27.53±3.346 μM for 24 h treatment while 24 h treatment of 10 μM fanchinoline could inhibit about 50% of the enzyme activity of cellular proteasome β1 subunit. Furthermore, accumulation of ubiquitinated proteins in fangchinoline-treated cells could be observed at dose as low as 8 μM for 24 h treatment or after only 1 h treatment of 27 μM fangchinoline. These results suggested the involvement of proteasome inhibition in cytotoxicity of fangchinoline. The inhibiting effects of fanghinoline on C-L activity were also observed in purified human 20S proteasome. However, the Ki of fangchinoline, 356.58±30.63 μM, in inhibiting activity of purified 20S proteasome was much higher than the dose needed for inhibiting cellular proteasome activity. The results indicated that the inhibiting effects of fangchinoline on cellular proteasome activity might include both direct effects on catalytic subunits as well as effects on other regulative components of proteasome. The later might deserve further study in the future.

The successful use of proteasome inhibitors bortezomib[35] and carfilzomib[36] in clinic suggested the important role of proteasome inhibitors in anti-cancer therapy. Many compounds including natural products such as marizomib (also known as NPI-0052), celastrol, and EGCG were in clinical or pre-clinical stages[37]. While, both bortezomib and cartizomib were molecules with inhibition mainly on CT-L activity which was mediated by proteasome β5 subunit[38]. Up to now, the role of β1 or β2 subunit in cancer therapy and the possibility of developing molecules with selective inhibition on the C-L or T-L activity of proteasome into new anti-cancer agents are still not clear. In the present study, fangchinoline and tetrandrine were found to be inhibitors specifically act on proteasome β1 subunit but not β2 or β5 subunit. We then further studied the involvement of proteasome inhibition in the anti-cancer effects of fangchinoline. Consistent with previous results[2, 11, 13], fangchinoline was found to induce G0/G1 phase arrest and apoptosis in PC-3 cells. And, in nude mice inoculated with PC-3 cells, fangchinoline dose-dependently inhibited tumor growth and induced apoptosis in tumor xenografts. Importantly, inhibition of proteasome activities by fangchinoline was also observed in tumor xenografts of fangchinoline-treated animals.

The cell cycle arrest and apoptosis induced by fangchinoline might be resulted from accumulation of proteasome substrates which played important roles in cell cycle progression and apoptosis. Time-dependent and dose-dependent accumulation of ubiquitinated proteins were observed in PC-3 cells treated with fangchinoline. Specifically, fangchinoline induced accumulation of Ub-p27, Ub-Bax and Ub-IκB-α. These results suggested that fangchinoline could inhibit degradation of p27, Bax and IκB-α. p27 could directly inhibit the activities of CyclinE/Cdk2 which promotes S phase transition, and CyclinD/Cdk4,6 which promotes cell cycle progression in early G1 to late G1[39]. Therefore, p27 plays a pivotal role in the control of G0/G1 to S phase transition during cell cycle progression. In the present study, the G0/G1 cell cycle arrest induced by fangchinoline might be resulted from the considerable increase in p27 and subsequent negative control of the cell cycle progression through G0/G1- to S- phase[40]. Our results were consistent with previous reports about increased level of p27 and G0/G1 arrest in fangchinoline-treated cells[13, 27]. Notably, it had been shown that cell cycle-dependent caspase-like activity that cleaves p27 is the β1 subunit of the 20S proteasome[41]. The specific role of β1 subunit in direct binding and degrading of p27 was further confirmed in a recent study[39]. Our results that finding fangchinoline as a proteasome β1 subunit inhibitor provided explanation for increase of p27 level and G0/G1 arrest in cells treated with fangchinoline. Furthermore, tetrandrine, which was also found to be a proteasome β1 subunit inhibitor in the present study, was also reported to increase p27 level and induce G0/G1 arrest in cancer cells[18–22, 27, 42]. Bax is a well known protein involved in regulation of apoptosis and proteasome inhibitors were reported to induce apoptosis by regulating degradation of Bax[28, 43–45]. Increased in Bax level of fangchinoline-treated cells were found in previous reports[2, 13, 46] as well as in the present study. The effects of fangchinoline on Bax might contribute to apoptosis induced by fangchinoline. IκB-α is a critical regulative factor of NF-κB pathway which plays role in cell proliferation[47, 48] as well as inflammatory response[49–51]. Inhibition on degradation of IκB-α might be involved in the effects of fangchinoline on cell proliferation. And, the possible involvement of accumulation of Ub-IκB-α in the anti-inflammatory effects of fangchinoline as well as that of Fangji might deserve further study.

In the present study, to confirm the role of proteasome inhibition in cytotoxicity of fangchinoline, both cells with over-expression of proteasome β1 subunit and cells with knockdown of proteasome β1 subunit were used. Cytotoxicity of fangchinoline was found to be enhanced in cells with over-expression of proteasome β1 subunit and ameliorated in cells with knockdown of proteasome β1 subunit. These results suggested that inhibition on proteasome β1 subunit might be involved in the cytotoxicity of fangchinoline. Furthermore, our results showed that knockdown of proteasome β1 subunit not only ameliorated apoptosis but also ameliorated autophagy induced by fanchinoline. Fangchinoline was reported to induce autophagy in other cancer cells[12] and tetrandrine also could induce considerable autophagy [52–54]. Since the ubiquitin-proteasome system and autophagy were closely related[55], it would be interesting in the future to further clarify the role of proteasome inhibition in the autophagy-inducing effects of fangchinoline as well as tetrandrine.

In summary, the main finding in the present study is clarification of the contribution of proteasome inhibition to the anti-cancer effects of fangchinoline. By inducing accumulation of proteasome substrates such as p27, Bax, and IκB-α, fangchinoline induced G0/G1 phase arrest and apoptosis in cultured cancer cells and also inhibited growth of tumor xenografts in nude mice. To be noted, fangchinoline was reported to have other activities such as inducing formation and conformational conversion of DNA G-quedruplexes[56] and reversing multidrug resistance by inhibiting P-glycoprotein activity in multidrug resistant human cancer cells[57]. Therefore, proteasome inhibition might be one of the mechanisms of the anti-cancer effects of fangchinoline.

## Supporting Information

S1 Table Data of tumor volume (mm^3^) of each group in nude mice experiment. (DOCX)
