# Supplementary material for: Inhibition on Proteasome β1 Subunit Might Contribute to the Anti-Cancer Effects of Fangchinoline in Human Prostate Cancer Cells
Source: PLoS One. 2015 Oct 29;10(10):e0141681. doi: 10.1371/journal.pone.0141681 (PMC4626104; doi:10.1371/journal.pone.0141681)
Supplement: S1 Table — (DOCX) [file pone.0141681.s001.docx]

**Supplemental S1 Table Data of** **tumor volume (mm^3^) of each group in nude mice experiment**

| Day after inoculation | Vehicle control (Mean ± SEM) | Vehicle control (Maximum) | Fanchinoline 25mg/kg (Mean ± SEM) | Fanchinoline 25mg/kg (Maximum) | Fanchinoline 50mg/kg (Mean ± SEM) | Fanchinoline 50mg/kg (Maximum) | Docetaxel (Mean ± SEM) | Docetaxel (Maximum) |
| --- | --- | --- | --- | --- | --- | --- | --- | --- |
| 14 | 322.0±26.21 | 404.51 | 370.0±42.37 | 519.59 | 348.4±3.470 | 356.43 | 356.6±27.74 | 439.83 |
| 17 | 608.9±56.24 | 834.72 | 356.5±51.63 | 512.11 | 321.3±21.46 | 402.14 | 415.1±26.02 | 510.42 |
| 21 | 1024±139.1 | 1612.12 | 577.7±53.05 | 812.68 | 374.8±37.58 | 462.36 | 368.4±32.93 | 505.23 |
| 24 | 1288±166.3 | 1889.22 | 955.0±78.87 | 1147.52 | 593.8±593.8 | 812.28 | 383.0±35.29 | 493.84 |
| 27 | 1520±148.9 | 2065.71 | 1248±50.73 | 1439.56 | 857.0±41.00 | 1008.40 | 377.0±46.11 | 573.23 |
| 30 | 2162±169.1 | 2706.25 | 1413±61.89 | 1655.05 | 1120±66.28 | 1263.09 | 369.3±71.32 | 689.19 |
